# Vitamin D Deficiency Predicts the ST Elevation Type of Myocardial Infarction in Patients with Acute Coronary Syndrome

**Published:** 2018

**Authors:** Naser Safaie, Haleh Rezaee, Babak Seif Dvati, Taher Entezari-Maleki

**Affiliations:** a *Cardiovascular Research Center, Tabriz University of Medical Sciences, Tabriz, Iran. *; b *Department of Clinical Pharmacy, Tabriz University of Medical Sciences, Tabriz, Iran.*

**Keywords:** Vitamin D deficiency, Myocardial infarction, Acute coronary syndrome, STEMI, Non-STEMI

## Abstract

According to studies, a significant association exists between the low levels of vitamin D and cardiovascular diseases such as myocardial infarction (MI). In a prospective case control study, 88 patients with acute coronary syndrome (ACS) including ST elevation myocardial infarction (STEMI) and Non-STEMI were enrolled. The plasma level of 25-hydroxy vitamin D [25(OH) D] was obtained at the time of acute MI. To assess the association between study variables logistic regression analysis was done. The overall rate of vitamin D deficiency was documented in 59.1% with the significantly higher prevalence rate in STEMI group (77.5% versus 43.7%; *p* = 0.001). In STEMI group, the plasma level of 25(OH) vitamin D was significantly lower than non-STEMI group (13.5 ± 7.7 versus 24.3 ± 14.9; *p* = 0.001). Vitamin D deficiency was the main predictor in occurring the ST elevation type of MI (Odd ratio: 8.1, 95% CI: 2.3 – 28.2; *p* = 0.001). The results of the present study demonstrated a higher prevalence of vitamin D deficiency among ACS patients. Furthermore, vitamin D deficiency was responsible for occurring ST elevation type of MI among ACS patients. Large studies are needed to confirm these findings.

## Introduction

Vitamin D deficiency is a common problem with a worldwide prevalence (1). Recent observational studies indicated that vitamin D deficiency may be the underlying cause of most health issues and diseases (1). According to studies, a significant association exists between the low levels of vitamin D and cardiovascular diseases. It is reported that vitamin D deficiency is associated with risk of hypertension, diabetes, hyperlipidemia, peripheral artery disease, heart failure, coronary artery disease, myocardial infarction (MI), and stroke. Accordingly, vitamin D deficiency is considered as a primary risk factor for cardiovascular diseases (1-6). 

Vitamin D receptors (VDRs) are present in most tissues including vascular smooth muscle, endothelium, and myocytes. The active form of vitamin D (1,25–dihydroxy vitamin D or calcitriol) inhibits rennin secretion, proliferation of vascular smooth muscles and myocytes and regulates cell growth. On the other hand, calcitriol has inhibitory effects on the secretion of some cytokines from lymphocytes. Vitamin D receptor agonists (VDRAs) inhibit the development of atherosclerosis, calcification of the arteries, heart muscle hypertrophy, thrombosis, and suppress renin-angiotensin system by acting on these receptors (1-6). 

Given the significant link of vitamin D deficiency with cardiovascular disease and molecular mechanisms of vitamin D, this study was aimed to evaluate the association between vitamin D deficiency and occurring of ST-elevation type of myocardial infarction in patients with acute coronary syndrome (ACS). 

## Experimental

*Design and Setting*

In a prospective case control study, all patients with acute myocardial infarction were enrolled from October to November 2014 in Shahid Madani Heart Center (SMHC), the largest university affiliated referral hospital for cardiovascular disorders at thenorthwest of Iran. The study was approved in the Ethic committee of the university. All patients filled the approved informed consent form. 

*Sample size calculation*

According to the first type error α = 0.05 and statistical power of 95% and also clinical studies power of 80%, as well as the prevalence of vitamin D deficiency in our country (an average of 75%), a minimum sample size of 18 patients was calculated to enter the study.

*Study Population*

All of the consented patients with 18 years old and over, with diagnosis of acute MI including both ST-segment elevation of MI (STEMI) and Non ST-segment elevation of MI (NSTEMI), were enrolled to the study. The study population was randomly selected from both STEMI and NSTEMI. The systematic randomization using computer generated random numbers was done for sampling. The exclusion criteria included the existent of autoimmune and inflammatory diseases, pregnancy, acute and chronic renal and liver dysfunction and the recent use of vitamin D supplements during last one month. 

*Blood sampling and 25 hydroxy vitamin D measurements*

Blood sampling for plasma levels of 25-hydroxyvitamin D 25(OH) D was obtained at the time of acute MI. Serum levels of 25-hydroxyvitamin D was measured to compare with severity of acute MI. We used Enzyme- Linked Immune-Sorbent Assay (ELISA) to measure 25-hydroxy vitamin D level with a Microplate Absorbance Reader (2020 sw-version 2.0 up) and factory kit (Euroimmun, Germany). The minimum measurable level of vitamin D in this method was 0 ng/mL. Measuring range was 0 -120 ng/mL and the normal range predicted by the kit was 30-100 ng/mL.

Vitamin D deficiency was defined as serum levels below 30 ng/mL of 25-hydroxyvitamin D. After considering the vitamin D deficient patients, they were classified into three groups according to serum 25-hydroxy vitamin D levels. Group A was reported with vitamin D level of 15-30 ng/mL as mild to moderate deficient, group B with 15-10 ng/ mL as severe deficient and group C, with vitamin D level of 10 ng/mL and lower as very severe vitamin D deficient (1).

*Statistical analysis*

The data were analyzed using SPSS version 16.0 (Chicago, SPSS Inc., 2007). Kolmogorov-Smirnov test was used to assess normally distribution of the data. Paired T-test or Wilcoxon were used for comparison of variables between two groups. Independent t-test or Mann-Whitney U Test was also used to compare variables between two groups. The Chi-square and Fisher’s exact test was used to compare the non-quantitative data (frequency) between the two groups. Correlation of variables was done with Spearmanꞌs rank-order correlation coefficient. To assess the association between study variables Logestic regression analysis was also done. In all analyses, P-value of less than 0.05 was considered to be statistically significant.

## Results

After a 6-month study period, the total numbers of 88 patients were eligible to enter the study. STEMI group contained 40 patients along with 48 patients in Non-STEMI group. Most of the patients in both group were male (n = 25 in STEMI and n = 36 in the other group). The mean ± SD for age in STEMI group was 59.2 ± 8.9 and 57.1 ± 9.4 in Non-STEMI group (Table 1).

In the present study, the overall rate of vitamin D deficiency was documented in 59.1% with the significantly higher prevalence rate in STEMI group (77.5% versus 43.7%; *p* = 0.001). Also, the prevalence of vitamin D insufficiency was 19.3% (n = 17) (15% in STEMI group versus 22.9% in non-STEMI group) (Table 2). In STEMI group, the plasma level of 25(OH) vitamin D significantly was lower than non-STEMI group (13.5 ± 7.7 versus 24.3 ± 14.9; *p* = 0.001).

In correlation analysis using Spearman test, a negative correlation between the incidence of STEMI and the level of 25-hydroxy vitamin D (*r* = -0.416; *p* = 0.0001) and diabetes (*r* = -0.368; *p* = 0.0001) were observed. The linear regression analysis showed two models linking independent factors of the study with STEMI. Based on these regression models, there is a negative association between the level of vitamin D and diabetes with occurring of STEMI (Table 3).

On the other hand, data analysis showed that the most diabetic patients experienced the non-STEMI (six diabetic cases in STEMI group versus 20 diabetic cases in non-STEMI group, 

*p *= 0.003).

The analysis of logistic regression revealed that the status of vitamin D deficiency (Odd ratio: 8.1, 95% CI: 2.3 – 28.2; *p* = 0.001) and history of previous MI (Odd ratio: 7.9, 95% CI: 1.5 – 42; *p* = 0.015) are the main predictors in occurring the STEMI. 

## Discussion

The present study was planned to evaluate the association between the level of 25-hydroxy vitamin D and the type of acute MI. based on the results, vitamin D deficiency could predict the STEMI. On the other hand, vitamin D deficiency was associated with the severe type of MI presenting with ST segment elevation on electrocardiography.

**Table 1:** Demographic data of the patients.* SD: standard deviation

**Demographic/clinical**	**STEMI group** **N = 40**	**Non-STEMI** **N = 48**	**P-value**
Age (years), mean ± SD	59.2 ± 8.9	57.1 ± 9.4	0.28
Male, n (%),	25 (62.5)	36 (75)	0.21
Female, n (%)	15(37.5)	12(25)	0.21
Weight (Kg), mean ± SD	85.6 ± 8.2	80.2 ± 7.5	0.02
Male Age, mean ± SD	59 ± 8.4	56.7 ± 9.8	0.328
Female Age, mean ± SD	59.4 ± 10.2	53.2 ± 16.3	0.234
Male 25-hydroxy vitamin D ( ng/mL), mean ± SD	13.3 ± 7.5	26.5 ± 15.5	0.0001
Female 25-hydroxy vitamin D (ng/mL), mean ± SD	14 ± 8.2	17.7 ± 10.8	0.323
Serum creatinine (mg/dL), mean ± SD	1.06 ± 0.2	1.06 ± 0.2	0.97
Blood Urea Nitrogen (mg/dL), mean ± SD	20.1 ± 5.8	18.7 ± 4.7	0.33
Hemoglobin (g/dL), mean ± SD	13.7 ± 2.1	13.4 ± 1.7	0.46
Fasting blood glucose (mg/dL), mean ± SD	118.7 ± 35.7	136.8 ± 45.5	0.02
Plasma 25 (OH) vitamin D (ng/mL), mean ± SD	13.5 ± 7.7	24.3 ± 14.9	0.001

**Table 2:** Patients’ medical and drug history

**Medical and drug history**	**STEMI (n = 40)**	**Non-STEMI (n = 48)**	***P*** **-value**
Vitamin D deficiency, n (%) Vitamin D insufficiency, n (%)	31 (77.5) 6 (15)	21 (43.7) 11(22.9)	0.001 0.349
Diabetes mellitus, n (%)	6 (15)	20 (41.6)	0.003
Myocardial infarction, n (%)	10 (25)	6 (12.5)	0.130
Hypertension, n (%)	23 (57.5)	26 (54.1)	0.754
Dyslipidemia, n (%)	14 (35)	20 (41.6)	0.522
Other disease, n (%)	3 (7.5)	1 (2.1)	0.326
Family history of cardiovascular disease, n (%)	7 (17.5)	16 (33.3)	0.092
Cardiovascular drugs, n (%)	21(52.5)	31 (64.6)	0.251
Anti-diabetic drugs, n (%)	6 (15)	20 (41.6)	0.003
Anti-lipid drugs, n (%)	17 (42.5)	24 (50)	0.483

**Table 3:** The linear regression models for determining independent factors associated with ST-elevation myocardial infarction

**Model**	**Factor**	**Standard Error**	**Beta**	***p*** **-value**	**95% Confidence Interval**	**R**	**R** ^2^
1	25-hydroxy vitamin D	0.006	-0.393	0.006	-0.028- -0.005	0.393	0.154
2	25-hydroxy vitamin D Diabetes mellitus	0.006 0.136	-0.364 -0.296	0.006 0.028	-0.027- -0.004 -0.582 - -0.035	0.491	0.241

In this study, a higher rate of vitamin D deficiency and insufficiency was documented among patients with acute MI that is in line with several epidemiologic and observational studies (1-6) and our previous works (7-9).

Several large studies have addressed the association of vitamin D deficiency and risk of coronary heart disease. 

In the Health Professionals Follow-up Study conducted on 18,225 men with a 10-year follow-up, it was shown that the vitamin D deficient men were at increased risk for development of MI compared with the vitamin D sufficient men (relative ratio: 2.42; 95% CI: 1.53-3.84; *p *< .001) which is consistent with our findings (3). 

Furthermore, based on the Framingham Offspring prospective study, with 1,739 participants, after a mean follow-up of 5.4 year, a significant association between low levels of vitamin D and the incidence of coronary heart disease was observed (HR: 1.81; 95% CI 1.03–3.18; *p *< 0.01) (2). 

The resent data from the MONICA/KORA Augsburg Case-Cohort Study on 1,783 German population with a mean follow-up period of 11 years suggested that higher vitamin D levels were linked with decreased risk of coronary heart disease especially in woman gender (10). 

In the Cardiovascular Health Study on 2,312 participants older than 65 years who were free of cardiovascular disease, it was shown that each 10-ng/mL decrease in vitamin D level caused a 9% (95% CI 2–17%) increase in risk of mortality and a 25% (95% CI: 8–44%) increase in risk of MI. Moreover, serum vitamin D concentrations < 15 ng/mL were linked with a 29% (95% CI: 5% to 55%) higher risk for mortality (11). 

The recent published systematic review and meta-analysis including 73 cohort studies and 22 randomized controlled trials with 880,128 participants showed a pooled relative risks of 1.35 (95% confidence interval 1.13 to 1.61) for death from cardiovascular disease in vitamin D deficient patients. Furthermore, vitamin D_3_, when given singly, reduced all-cause mortality significantly by 11%. However, the supplementation of vitamin D_2_ had no effect on overall mortality (12).

In contrary, some studies have failed to show the significant association between low vitamin D levels and coronary heart disease deaths. For example, based on data from MINI- Finland Health Survey on 6,219 men and women > 30 years old and free from cardiovascular disease, after adjustment for season and traditional cardiovascular risk factors no significant association was documented between vitamin D and coronary heart disease deaths after a median follow-up of 27 years (HR : 0.91; 95% CI 0.70 to 1.18; *p* = 0.20) (13).

The anti-atherosclerotic properties of vitamin D have been described well by Kassi *et al*. in a review article (14). Based on this review, vitamin D could active nitric oxide (NO) synthase in endothelial cells and hence could increase NO level as a vasodilator agent. Besides, reactive oxygen species (ROS) production was decreased by vitamin D. Vitamin D also acts as an anti-inflammatory agent by inhibition of interleukin-6 (IL-6), IL-8 and regulated on activation normal T cell expressed, and secreted (RANTES). Furthermore, it suppresses cyclo-oxygenase (COX)-2 expressions, and stimulates 15-hydroxyprostaglandin dehydrogenase (15PGDH) production (the enzyme initiating prostaglandin (PG) catabolism). Vitamin D also inhibits adhesion cell molecules mainly by a nuclear factor-κB (NF-κB)–mediated mechanism such as E-selectin, intercellular adhesion molecule-1 (ICAM-1), vascular cell adhesion molecule-1 (VCAM-1), platelet endothelial cell adhesion molecule-1 (PECAM-1) that can result in decreasing thrombosis formation. Vascular tonicity could be also acutely regulated by vitamin D through reducing calcium influx into the endothelial cells (ECs) and hence decreasing the production of endothelium-derived contracting factors (EDCFs). Furthermore, vitamin D directly can downregulate COX-1 as a main source of EDCFs.

Vitamin D also indirectly exerts anti-atherosclerotic properties by combating insulin resistance, β-cell dysfunction, dyslipidemia, and the rennin-angiotensin-aldosterone system (RAAS) (14). 

Taken together, our study findings are in line with many clinical observations linking vitamin D deficiency with cardiovascular diseases and showed that the probable incidence of STEMI in vitamin D deficient clients was about 8 times more than other ACS patients. As mentioned above, this finding was elucidated by the molecular mechanisms of vitamin D and accordingly by clinical observations. However, some studies also failed to show the clinical association between vitamin D and cardiovascular diseases. Importantly, there is no clear evidence about the effect of supplementing of vitamin D in cardiovascular diseases besides a number of data has shown a controversial result. These observations may be attributable to existing of some discrepancies in the setting and method of studies, population, and genetic differences, dose and duration of vitamin D therapy. Large trials are still needed to put a clear role for vitamin D in therapeutic process of cardiovascular diseases. But, currently what is clear is the supplementing vitamin D to prevent and to treat vitamin D deficient individuals who are at risk of cardiovascular diseases. 

*Study limitations*

This study like the other clinical studies may have some limitations. First, despite calculating sample size, this study has partially small sample size. Second, we encountered with cost limitations. Third, study of more disease related variables such as cardiac biomarkers, inflammatory cytokines, and echocardiography studies are recommended to clear understanding between the relation of vitamin D deficiency and coronary heart diseases. 

## Conclusion

The results of the present study demonstrated a higher prevalence rate of vitamin D deficiency among ACS patients. Furthermore, vitamin D deficiency was responsible for occurring of ST elevation type of MI among ACS patients. Large studies are needed to confirm these findings.

## Declaration of conflicts of interest

There are no conflicts of interest to declare. 

## Funding

This study did not receive funding from any sources.
